# Effect of Achievement Motivation and Self-Efficacy on General Well-Being among Students at Normal Universities in Ningxia: The Mediating Role of Time Management

**DOI:** 10.3390/bs14010015

**Published:** 2023-12-24

**Authors:** Jingyi Dong, Norlizah Che Hassan, Aminuddin Bin Hassan, Dan Chen, Wei Guo

**Affiliations:** 1 Faculty of Educational Studies, Universiti Putra Malaysia, Selangor 43300, Malaysia; djyooo2023@163.com (J.D.); aminuddin@upm.edu.my (A.B.H.); 2 Department of Foreign Language Teaching, Ningxia Medical University, Yinchuan 750000, China; 3 Faculty of Physical Education, Ningxia Normal University, Yinchuan 750000, China

**Keywords:** achievement motivation, self-efficacy, time management, general well-being, university students

## Abstract

General well-being is a positive evaluation of one’s mental health, which is an important topic in mental health. General well-being is fundamental to the positive development of young people. A thorough understanding of the factors that influence happiness have not yet been developed for students at normal universities in Ningxia Province in China. This study examined the mediation effects of time management on the relationship between achievement motivation, self-efficacy, and general well-being among students at normal universities in Ningxia Province in China. Using a random sampling, 163 participants (68 males and 95 females) completed the Achievement Motivation Scale (AMS), General Self-efficacy Scale (GSES), Time Management Questionnaire (TMQ) and General Well-being Scale (GWBS). Achievement motivation, self-efficacy, and time management were positively correlated with general well-being. The mediation effects of time management on the relationships between achievement motivation, self-efficacy, and general wellbeing (0.169 and 0.447) were demonstrated. These results add to the nuanced relationship between self-efficacy, achievement motivation, and general well-being. According to the Achievement Motivation Theory and the Self-efficacy Theory, this study reveals the role of achievement motivation, self-efficacy on general well-being and the mediating effect of time management in the relationship between achievement motivation, self-efficacy, and general well-being. According to the findings, the school can carry out a series of lectures and tutoring activities to enhance students’ achievement motivation, self-efficacy, time management, and general well-being.

## 1. Introduction

General well-being has been defined as the experience of pleasant emotions like happiness and satisfaction, and the ability to feel good and operate well, the development of personal potential, having some control over life, having goals, and experiencing positive connections [1]. General well-being is a positive evaluation of one’s mental health, which is an important topic. Individuals with high life satisfaction and high positive emotions may have less depression, loneliness, higher self-esteem, and fewer mental problems [2].

At present, the incidence of psychological problems among college students is frighteningly high, among which depression, anxiety, stress, and other diseases are the most common, accounting for 76%, 88.4%, and 45.5% [3,4,5]. These issues can affect the general well-being of college students [6]. When college students enter university, they often feel academic tension, which can also have a certain impact on their general well-being [7]. Among college students, academic stress can increase due to heavy workloads, inadequate faculty support, and an unsupportive campus environment [8,9]. In addition, academic performance is a major factor in college students’ employment success. It makes some students feel more stress, resulting in anxiety, depression, and so on [10,11,12]. The lack of quality of life may lead to some negative consequences, such as psychological stress, physical discomfort, low mood, social isolation, etc. [13,14]. The evaluation of college students’ general well-being has become a key issue in current social and mental health research [15].

Students in normal universities are the key group for the cultivation of educational talents. The term “normal university” is a kind of oxymoron, or contradiction of terms. The term “normal” in English can only be clearly understood with reference to its French roots, where it means “setting a moral standard or pattern” [16]. Students from normal Universities in Ningxia will become teachers, educational administrators, and educational researchers in the future. Their educational professional ability will directly affect the development and promotion of education in Ningxia. Ningxia is in the stage of rapid development and transformation, requiring both social stability and sustainable development. University students in normal universities have a professional educational background and knowledge, making them a crucial force in promoting social development in Ningxia [17].

According to the Achievement Motivation Theory and the Self-efficacy Theory, achievement motivation and self-efficacy can influence general well-being [18,19,20,21,22,23,24,25]. In addition, some studies have showed that time management as well can influence general well-being. Therefore, the current study was aimed to identify the relationship among achievement motivation, self-efficacy, and time management towards general well-being of students at normal universities in Ningxia Province in China.

Motivation refers to an innate drive that motivates people to engage in certain activities, and achievement motivation is an important form of motivation. A person’s success depends on his motivation [18]. In the context of the knowledge economy, motivation is a very important psychological element that occupies a central position among non-intellectual elements and exerts a significant influence on it [19]. Achievement motivation is the self-motivating force created by exceptional people in the survival of the fittest struggle. People are driven to want to engage in tough and meaningful activities, and to be better than others by this motivation [20].

According to the Achievement Motivation Theory, when an individual is able to achieve personal goals, overcome challenges, and achieve success, he will experience a positive emotion, thus improving well-being. Individuals with high achievement motivation are generally more determined and motivated to pursue success, they value their own achievements more, and they have a higher likelihood of experiencing satisfaction and high general well-being [21].

Research shows that there is a strong relationship between people’s achievement motivation and their level of general well-being. Numerous studies have shown that general well-being and achievement motivation are closely associated, and that high achievement motivation is essential for improving general well-being [22,23,24]. People with higher achievement motivation have higher needs for self-realization in their lives. As people progress towards self-realization, they will have a higher sense of well-being overall [23,24].

The Self-efficacy theory holds that an individuals’ beliefs and confidence allows them to effectively complete various tasks, namely self-efficacy, and it has an important impact on happiness. Individuals with higher self-efficacy feel they can take on challenges, get through obstacles, and accomplish goals, and are therefore more likely to experience the pleasure of achievement and have high general well-being. Contrarily, individuals with low self-efficacy frequently feel helpless and depressed, and lack confidence in achievement, which affects their general well-being [25].

Research has found that generalized self-efficacy is strongly associated with positive affects [26] and negative affects [27]. In addition, individual self-efficacy is also a key factor in improving individual general well-being [28].

Time management belongs to a common concept in every management field, and it describes a set of actions used to raise people’s time utilization rate, so that they can make better use of their time, so as to achieve their own or organizational goals [29]. People with high achievement motivation often have a strong desire for success and have a high enthusiasm for success. In order to achieve success, they need to understand the importance of time, monitor and use time reasonably and efficiently to achieve their goals. On the contrary, individuals with low levels of achievement motivation have insufficient understanding of time value and a low ability of time monitoring. Achievement motivation can predict time management tendency. Lu (2018) and Xin et al. (2020) have proved that the higher the college students’ achievement motivation, the stronger their time management ability and ability to effectively monitor time [30,31].

Zhang et al. (2011) and Li et al. (2021) pointed out that general self-efficacy can significantly positively predict time management tendency [32,33]. Individuals who have a high sense of general self-efficacy are full of confidence in their ability to deal with things, and they can deal with and arrange various problems properly, so they can better plan, arrange, and make use of their time. While time control is a common problem among college students, time control is also a recurring problem that can have an emotional and physical impact on students in rigorous majors [34,35,36,37]. Existing studies have shown that young people experience increased levels of stress and decreased levels of general well-being due to learning, environmental factors, and time-management skills [37,38,39,40]. Some previous independent studies have confirmed the effectiveness of some interventions that aim to increase general well-being in education environments, such as smart devices, and the use of time management aides [41,42,43,44,45]. Based on the results of previous research, hypotheses 1–5 were proposed.

**Hypothesis** **1.** *There is a positive influence of achievement motivation on general well-being.*

**Hypothesis** **2.** *There is a positive influence of self-efficacy on general well-being.*

**Hypothesis** **3.** *There is a positive influence of time management on well-being.*

**Hypothesis** **4.** *Time management mediates the relationship between achievement motivation and well-being.*

**Hypothesis** **5.** *Time management mediates the relationship between self-efficacy and well-being.*

## 2. Materials and Methods

Respondents in this study were students at normal universities in Ningxia. 215 questionnaires were collected online through Questionnair Star (a platform that randomly distributes questionnaires), then, 52 invalid cases (due to students from non-normal universities, and some respondents provided the same responses for a majority of the items in the questionnaires) were deleted, therefore, a total of 163 valid questionnaires were kept. According to the results of the demographic survey, the participants comprised 68 men (41.7%) and 95 women (58.3%). Among them, there were 59 freshman (36.2%), 21 sophomore (12.9%), and 83 three-grade students (50.9%).

We measured achievement motivation using the Achievement Motivation Scale (AMS) [46]. There are two dimensions of achievement motivation: pursuing success (15 items) and avoiding failure (15 items). The five-point Likert scale was used. Numerical numbers were given to represent the level of agreement of each item (1 “Strongly disagree” to 5 “Strongly agree”). The total score is obtained by subtracting the score of the avoiding failure dimension from the score of the pursuing success dimension. A higher mean on this scale indicates higher achievement motivation while a lower mean indicates lower achievement motivation. Test–retest reliability of the Achievement Motivation Scale is 0.77.

We evaluated students’ self-efficacy using the General Self-efficacy Scale (GSES) with 10 items [47]. The four-point Likert scale was used. Numerical numbers were given to represent the level of agreement of each item (1 “totally false” to 4 “totally true”). The coefficient of internal consistency of the General Self-efficacy Scale is 0.91.

We measured time management using the Time Management Questionnaire (TMQ) [48]. There are three dimensions of time management in this study: time value (15 items), time monitoring (19 items), and time efficacy (10 items). Among them, there are 5 negative items. The five-point Likert scale was used. Numerical numbers were given to represent the level of agreement of each item (1 “Strongly disagree” to 5 “Strongly agree”). A higher mean on this scale indicates higher time management while a lower mean indicates lower time management. Negative items were reversed and implemented by assigning the highest value (e.g., 5) to the lowest response and the lowest value (e.g., 1) to the highest response. The test–retest reliability of the Time Management Questionnaire and its dimensions is between 0.71 and 0.85, and the Cronbach Alpha value is between 0.61 and 0.81.

We measured general well-being using the General Well-being Scale (GWBS) (Dai, 2010) [49]. There are six dimensions of general well-being in this study: friendship (8 items), freedom (5 items), achievement (6 items), family (7 items), environment (5 items), and school (6 items). There are 5 negative items. The seven-point Likert scale was used. Numerical numbers were given to represent the level of agreement of each item (1 “Strongly disagree” to 7 “Strongly agree”). A higher mean on this scale indicates higher general well-being while a lower mean indicates lower general well-being. The coefficient of stability of the General Well-being Scale and its dimensions is between 0.67 and 0.82, and the Cronbach Alpha value is between 0.71 and 0.91.

Written informed consent was obtained from all subjects involved in the study. All respondents were informed that the information they provide, and their identity remain confidential.

Descriptive statistics, a *t*-test, an ANOVA, and a correlation analysis of the variables were all performed using SPSS 23.0. The SPSS PROCESS macro was used to the test hypotheses [50].

## 3. Results

### 3.1. Descriptive Statistics

As Table 1 shows, the overall mean of achievement motivation was 0.16 (SD = 0.8), meaning that there is little difference in the average score between pursuing success and avoiding failure. The overall mean of self-efficacy (1 “totally false” to 4 “totally true”), time management (1 “Strongly disagree” to 5 “Strongly agree”), and general well-being (1 “Strongly disagree” to 7 “Strongly agree”) obtained was 2.68 (SD = 0. 4), 3.51 (SD = 0.39), and 4.98 (SD = 0.71), respectively, meaning that the respondents’ self-efficacy, time management, and general well-being was positive and moderate.

### 3.2. Differences of Demographic Variables in Achievement Motivation, Self-Efficacy, Time Management, and General Well-Being

In order to identify differences in students’ achievement motivation, self-efficacy, time management, and general well-being between genders, an independent samples *t*-test was employed.

As shown in Table 2, the mean of the males’ achievement motivation was 0.099 (SD = 0.819), and the mean of the females’ achievement motivation was 0.207 (SD = 0.785). There were no significant differences in achievement motivation between genders (t = −0.851, *p* = 0.396 > 0.05). The mean of the males’ general self-efficacy was 2.71 (SD = 0.451), and the mean of the females’ general self-efficacy was 2.67 (SD = 0.355). There were no significant differences in general self-efficacy between genders (t = 0.602, *p* = 0.548 > 0.05). The mean of the males’ time management was 3.53 (SD = 0.431), and the mean of the females’ time management was 3.5 (SD = 0. 368). There were no significant differences in time management between genders (t = 0.554, *p* = 0.58 > 0.05). The mean of the males’ general well-being was 4.96 (SD = 0.736), and the mean of the females’ general well-being was 5 (SD = 0.696). There were no significant differences in general well-being between genders (t = −0.39, *p* = 0.697 > 0.05).

In order to identify differences in students’ achievement motivation, self-efficacy, time management, and general well-being between grades, ANOVA was employed.

As shown in Table 3, the mean of the freshmen’s achievement motivation was 0.1 (SD = 0.843), and the mean of the sophomore’s achievement motivation was −0.12 (SD = 0.597), and the mean of the three-grade students’ achievement motivation was 0.28 (SD = 0.796). There were no significant differences in achievement motivation between grades (F = 2.392, *p* = 0.095 > 0.05). The mean of the freshmen’s self-efficacy was 2.61 (SD = 0.305), and the mean of the sophomore’s self-efficacy was 2.69 (SD = 0.418), and the mean of the three-grade students’ self-efficacy was 2.73 (SD = 0.444). There were no significant differences in general self-efficacy between grades (F = 1.499, *p* = 0.226 > 0.05). The mean of the freshmen’s time management was 3.5 (SD = 0.368), and the mean of the sophomore’s time management was 3.54 (SD = 0. 476), and the mean of the three-grade students’ time management was 3.51 (SD = 0.395). There were no statistically significant differences in time management between grades (F = 0.114, *p* = 0.893 > 0.05). The mean of the freshmen’s well-being was 5.12 (SD = 0.73), and the mean of the sophomore’s well-being was 4.86 (SD = 0. 841), and the mean of the three-grade students’ self-efficacy was 4.91 (SD = 0.653). There were no statistically significant differences in general well-being between grades (F = 1.875, *p* = 0.157 > 0.05).

### 3.3. Relationships between Students’ Achievement Motivation, Self-Efficacy, Time Management, and Well-Being

In this study, Pearson’s correlation was chosen to represent the relationship between students’ achievement motivation, general self-efficacy, time management, and general well-being.

Table 4 shows that the correlations are all significant, and the values are between 0.250 and 0.545. The value of the Pearson Correlation between achievement motivation and general well-being was 0.250, so there was a significant low positive relationship between achievement motivation and general well-being. The result revealed that students who have high achievement motivation tend to have high general well-being. The table shows that the relationship between self-efficacy and general well-being was a significant low positive relationship as well (r = 0.252, *p*= 0.001). The result implied that students with high self-efficacy tend to have high general well-being. The results from the Pearson’s Correlation statistics analysis showed that there was a significant moderate relationship between time management and general well-being (r = 0.545, *p* < 0.001). The result also implied that students who have high time management tend to have high general well-being.

### 3.4. Hypotheses Tests

The regression results (Table 5) show that achievement motivation, and time management can predict general well-being. In model 1, achievement motivation can significantly positively predict general well-being (β = 0.250, *p* < 0.001). Model 2 showed that there was a significant positive effect between achievement motivation and time management (β = 0.352, *p* < 0.001). The results of model 3 showed that there was a positive effect between achievement motivation and general well-being (β = 0.066, *p* > 0.05), and time management significantly predicted general well-being (β = 0.522, *p* < 0.001). In model 3, after time management was added, the effect of achievement motivation on general well-being was not significant.

The regression results (Table 6) show that self-efficacy, and time management can predict general well-being. In model 1, self-efficacy could significantly positively predict general well-being (β = 0.252, *p* < 0.01). Model 2 showed that there was a significant positive effect between self-efficacy and time management (β = 0.455, *p* < 0.001). The results of model 3 showed that there was a positive effect between self-efficacy and general well-being (β = 0.005, *p* > 0.05), and time management significantly predicted general well-being (β = 0.543, *p* < 0.001). In the model 3, after time management was added, the effect of self-efficacy on general well-being was not significant.

On the basis of the regression model, the mediation model of SPSS macro was used. The bootstrap method was conducted to examine if the mediating effect was significant; the sample size was 5000 and the confidence was 95%. Based on this, the mediating effect of time management between achievement motivation, self-efficacy, and general well-being was analyzed. Table 7 and Table 8 show the results.

From Table 7, the direct effect is 0.059, and the confidence interval of 95% is [−0.065, 0.183], including 0, pointing out that the effect of achievement motivation on general well-being is not significant. The total indirect mediating effect of time management is 0.164, and the confidence interval of 95% is [0.094, 0.237], excluding 0, pointing out that time management has a significant mediating effect on the relationship between achievement motivation and general well-being. It shows that there is full mediation. Full mediation would occur if inclusion of the mediation variable drops the relationship between the independent variable and dependent variable to zero [51,52,53].

**Figure 1:**
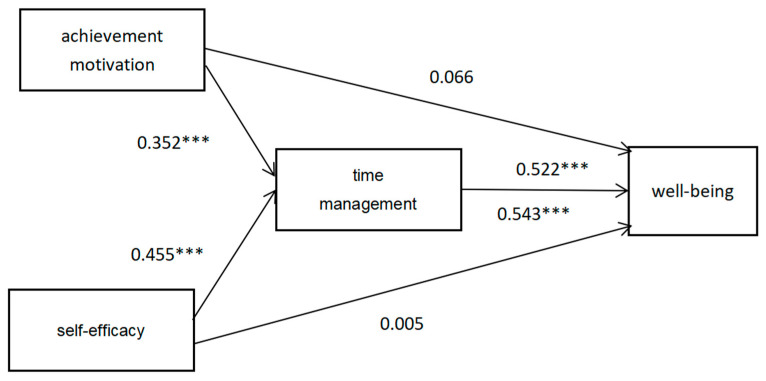
The mediation model with time management as a mediator of the linkage between achievement motivation, self-efficacy, and general well-being. *** *p* < 0.001.

From Table 8, the direct effect is 0.009, and the confidence interval of 95% is [−0.254, 0.272], including 0, pointing out that the effect of self-efficacy on general well-being is not significant. The total indirect mediating effect of time management is 0.442, and the confidence interval of 95% is [0.275, 0.613], excluding 0, pointing out that time management has a significant mediating effect on the relationship between self-efficacy and general well-being. It shows that there is full mediation as well. The specific path of achievement motivation and self-efficacy acting on general well-being through time management is detailed in Figure 1.

## 4. Discussion

As shown in Table 2, there were no statistically significant differences in achievement motivation, self-efficacy, time management, and general well-being between genders. This may be because male students and female students are under the same learning pressure, so there is no significant difference in time management. Although the role requirements and parenting styles of boys and girls are different in society and some families, on the whole, the status of boys and girls is relatively equal.

When students’ achievement motivation is strong, they often have the need to pursue success. In order to achieve success, they need to set corresponding goals. The achievement goal theory holds that goals play a guiding role and are an important dynamic mechanism for learners to achieve their goals. Goals will promote students to manage their time effectively [54]. Time management disposition can show the psychological and behavioral traits of people in how they use time. The sense of time monitoring allows individuals to reasonably arrange the priority of tasks according to their goals, allocate the main time to important learning tasks, and be good at disciplining themselves. The sense of time efficacy can help individuals evaluate their own time management ability. When individuals arrange their time reasonably and achieve their goals, they will have positive emotions and higher general well-being [55].

The relationships between self-efficacy, time management, and general well-being are amply supported by empirical data [56,57,58,59]. Therefore, we put forward the view that generalized self-efficacy can enhance individuals’ general well-being through time management. College students who have higher levels of general well-being and life satisfaction have stronger adaptability, resilience, and efficient ability to deal with problems, but also work harder to complete learning tasks and achieve more success [60]. This may be due to the fact that their strong self-efficacy beliefs may have an impact on their task selection, perseverance, effort, and academic success [22]. In essence, self-efficacy enhances higher commitment, problem-solving abilities, goal orientation, creativity, and time management skills [61,62], so as to improve general well-being [63].

## 5. Conclusions

The main purpose of the study is to examine the mediating role of time management in the effects of achievement motivation and self-efficacy on general well-being among students at normal universities in Ningxia. Based on the reviews of previous research and the purpose of the current study, five hypotheses has been proposed.

**Hypothesis** **1.** *There is a positive influence of achievement motivation on general well-being.*

**Hypothesis** **2.** *There is a positive influence of self-efficacy on general well-being.*

**Hypothesis** **3.** *There is a positive influence of time management on well-being.*

**Hypothesis** **4.** *Time management mediates the relationship between achievement motivation and well-being.*

**Hypothesis** **5.** *Time management mediates the relationship between self-efficacy and well-being.*

The findings showed that H1–H5 were all supported. Additionally, the data confirmed H4 and H5 by showing that time management had full mediation influence on the relationship between achievement motivation, self-efficacy, and general well-being.

## 6. Limitations and Implications

There are some restrictions in this study. First, the cross-sectional survey design could not test the causal relationships among variables. Therefore, a longitudinal design could be used as a follow-up survey method. Second, only self-reporting questionnaires were used in this study. In future investigation, different investigation methods need to be adopted in order to obtain more information. Lastly, the sample size of the current study is a bit small, and may lead to low sample representativeness.

Despite the limitations of this study, it has research value and significance. China has a large territory and a large population, and almost every region has its own characteristics. It is difficult to study every region at the same time, so every year there are a lot of studies of the areas in which the researchers belong. The studies of different regions can be compared with each other to understand the characteristics of each region. For example, some studies showed achievement motivation of college students in a certain city was positive and moderate, while some studies showed achievement motivation of college students in a certain city was very high. So, the present study adds to our understanding of the present level of achievement motivation, self-efficacy, time management, and general well-being among students at normal universities in Ningxia Province in China.

Achievement motivation, self-efficacy, time management, and general well-being are of great interest to researchers, and many researchers study these four variables together with other variables every year. But few researchers have studied these four variables together. Few studies put time management and happiness together, and generally time management and time insight, mobile phone dependence and so on are put together. So, the present study adds to our understanding of the relationship between achievement motivation, self-efficacy, time management, and general well-being. The study shows an interesting result that among achievement motivation, self-efficacy, and time management, time management was the most influencing predicting contributor to students’ general well-being.

The present study shows that achievement motivation, self-efficacy, time management, and general well-being among students at normal universities in Ningxia Province in China were not very high, and measures should be taken to improve them according to the situation.

The universities could hold lectures to communicate the importance and uniqueness of characteristic education in Ningxia, and help students understand the educational needs and challenges in Ningxia, so as to enhance their achievement motivation. In addition, students studying in normal universities can actively participate in educational projects and activities, such as education support projects, social practice, and so on. Through practical experience, they can improve their sense of self-efficacy and motivation and cultivate practical problem-solving abilities at the same time. In addition, social services could support people in their pain, loss, and anxiety, as well as their triumphs, joys, and victories, and enhance well-being [64,65,66].

According to the results of this study, time management has a significant correlation with general well-being, so if students’ time management skills are improved, their general well-being may also be improved. Good interpersonal relationships and excellent academic achievement are some of the important reasons for students’ happiness. Therefore, the school should provide tutoring to students who are not good at dealing with interpersonal relationships and want to improve their academic performance, so that they can acquire the corresponding skills. As students become more proficient in the application of their skills, students will become more adept at dealing with problems, and their self-confidence and self-efficacy will gradually increase. And when they acquire good interpersonal relationships and excellent academic achievement, their general well-being will increase.

## Figures and Tables

**Table 1:** Descriptive statistics.

	M	SD
achievement motivation	0.16	0.8
self-efficacy	2.68	0.4
time management	3.51	0.39
general well-being	4.98	0.71

**Table 2:** Independent samples *t*-test.

	Male (N = 68)	Female (N = 95)	t	*p*
	(M ± SD)	(M ± SD)		
achievement motivation	0.099 ± 0.819	0.207 ± 0.785	−0.851	0.396
self-efficacy	2.71 ± 0.451	2.67 ± 0.355	0.602	0.548
time management	3.53 ± 0.431	3.5 ± 0.368	0.554	0.58
general well-being	4.96 ± 0.736	5 ± 0.696	−0.39	0.697

**Table 3:** ANOVA.

	Freshman (N = 59)	Sophomore (N = 59)	Three-Grade Students (N = 83)	F	*p*
	(M ± SD)	(M ± SD)	(M ± SD)		
achievement motivation	0.1 ± 0.843	−0.12 ± 0.597	0.28 ± 0.796	2.392	0.095
self-efficacy	2.61 ± 0.305	2.69 ± 0.418	2.73 ± 0.444	1.499	0.226
time management	3.5 ± 0.368	3.54 ± 0.476	3.51 ± 0.395	0.114	0.893
general well-being	5.12 ± 0.73	4.86 ± 0.841	4.91 ± 0.653	1.875	0.157

**Table 4:** Correlations.

		1	2	3
1	achievement motivation	1		
2	self-efficacy	0.331 **		
3	time management	0.352 **	0.455 **	
4	general well-being	0.250 **	0.252 **	0.545 **

** *p* < 0.01.

**Table 5:** Hierarchical regression analysis results of achievement motivation, time management, and general well-being.

Variables	Model 1		Model 2		Model 3	
	General Well-Being		Time Management		General Well-Being	
	*β*	*t*	*β*	*t*	*β*	*t*
achievement motivation	0.250	3.278 **	0.352	4.779 ***	0.066	0.938
time management					0.522	7.386 ***
F	10.744 **		22.841 ***		34.432 ***	
R^2^	0.057		0.119		0.292	

** *p* < 0.01,***p < 0.001.

**Table 6:** Hierarchical regression analysis results of self-efficacy, time management, and general well-being.

Variables	Model 1		Model 2		Model 3	
	General Well-Being		Time Management		General Well-Being	
	*β*	*t*	*β*	*t*	*β*	*t*
self-efficacy	0.252	3.304 **	0.455	6.482 ***	0.005	0.069
time management					0.543	7.292 ***
F	10.916 **		42.011 ***		33.81 ***	
R^2^	0.058		0.202		0.288	

** *p* < 0.01,***p < 0.001.

**Table 7:** Effects and 95% confidence intervals of achievement motivation, time management, and general well-being.

Time Management	Achievement Motivation on General Well-Being			
	Effect	Boot SE	Boot LLCI	Boot ULCI
Direct effects	0.059	0.063	−0.065	0.183
Indirect effects	0.164	0.036	0.094	0.237

**Table 8:** Effects and 95% confidence intervals of self-efficacy, time Management, and general well-being.

Time Management	Self-Efficacy on General Well-Being			
	Effect	Boot SE	Boot LLCI	Boot ULCI
Direct effects	0.009	0.133	−0.254	0.272
Indirect effects	0.442	0.087	0.275	0.613

## Data Availability

The authors will freely share the unfiltered raw data that underlies the results of this article.
